# Aboriginal Australian weapons and human efficiency

**DOI:** 10.1038/s41598-024-76317-w

**Published:** 2024-10-26

**Authors:** Laura E. Diamond, Michelle C. Langley, Bradley Cornish, Claudio Pizzolato, David J. Saxby

**Affiliations:** 1 Australian Centre for Precision Health and Technology (PRECISE), Griffith University, Gold Coast, Australia; 2 School of Health Sciences and Social Work, Griffith University, Gold Coast, Australia; 3 Australian Research Centre for Human Evolution, Griffith University, Brisbane, Australia; 4 Archaeology, School of Environment and Science, Griffith University, Nathan, Australia

**Keywords:** Biomechanics, Kinetic energy, Kinematics, Indigenous, Conflict, Aboriginal Australia, Experimentation, Biomedical engineering, Skeleton, Archaeology

## Abstract

Aggression—and its role in human societal development—continues to be hotly debated within both the sciences and the humanities. Whatever the evolutionary origins and repercussions of interpersonal and intergroup conflict for the human story, cultures around the globe have invested significant time and effort into designing deadly hand-held weaponry. Here, we describe for the first time, how humans deliver a deadly strike using two iconic and widespread Aboriginal Australian weapons: the *kodj* and the *leangle* with parrying shield. We present the world’s first evaluation of striking biomechanics and human and weapon efficiency regarding this class of implement. Results demonstrate the *leangle* is far more effective at delivering devastating blows to the human body, while the *kodj*—a multi-functional tool—is more efficient for a human to manoeuvre and still capable of delivering severe blows that can cause death. Together, these data provide the beginnings of an in-depth understanding of how hand-held weaponry has impacted the human body throughout the deep past.

**Supplementary Information:**

The online version contains supplementary material available at 10.1038/s41598-024-76317-w.

## Introduction

Despite our capacity for empathy and cooperation, *Homo sapiens* have long engaged in acts of violence ranging from brief conflicts between individuals to long-term and state-based warfare^1–7^. It has even been hypothesised that the anatomical reconfiguration of the human hand from a more primate-like form with long, curved fingers and diminutive thumb to one featuring an opposable thumb with shorter, straighter fingers is tied to *Homo sapiens*’ increasing use of hand-held weapons^8^. Indeed, studies of modern chimpanzees have found that while this species will throw objects (rocks, sticks, leaves, etc.) in aggressive displays, they are generally not directed at others, nor do they use them to strike another individual directly^9,10^. As such, hand-held interpersonal weapons appear to be the exclusive domain of the human ape.

This legacy of interpersonal violence throughout human history and continuing into the present day has resulted in significant intellectual and physical effort being directed into the design of weapons which are thrown or swung^11,12^. Although a multitude of archaeological and forensic studies have been undertaken to understand and identify injuries to the human body resulting from interpersonal weapons (i.e.,^13^), in addition to the effectiveness of projectiles to penetrate animal and human bodies (e.g.,^14–17^), and war clubs to inflict lethal injuries^18^, the physical demands required of humans to strike effectively with interpersonal weapons remains unexamined. To understand exactly how humans deliver a deadly strike, it is necessary to understand both striking biomechanics and human and weapon efficiency.

**Fig. 1:**
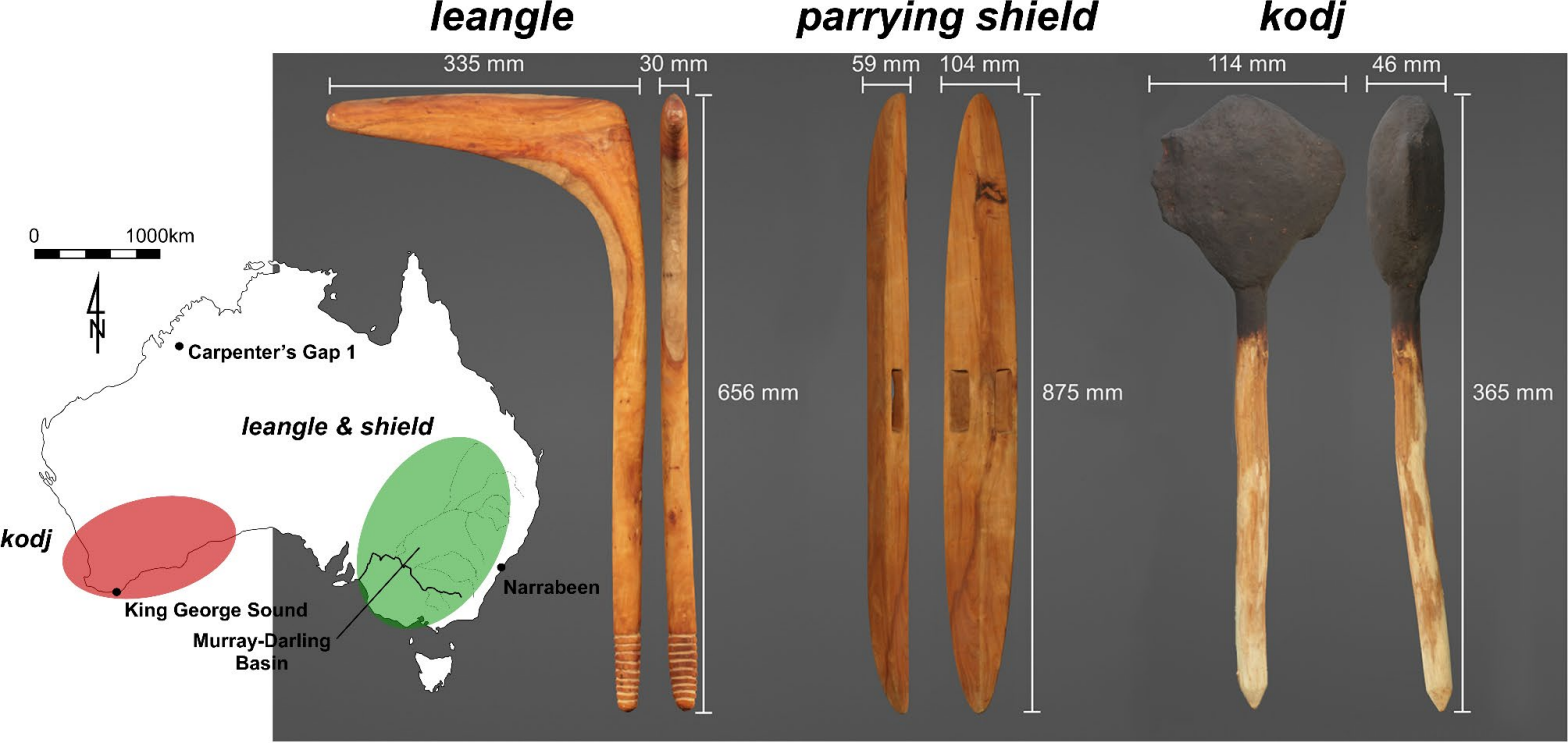
The *kodj* and *leangle* with parrying shield. Multi-view three-dimensional reconstruction and corresponding frontal and sagittal plane dimensions (mm) of the *leangle* (left) and parrying shield (centre) fabricated in southeastern Australia (Swan Hill, Victoria) is generally found in the green oval. The *kodj* (right) is found in the red oval with the example utilised in this experiment fabricated in southwestern Australia (Albany, Western Australia). The *leangle* and parrying shield are carved from hardwood. The *kodj* handle is carved from wattle wood, and a sharpened *boya* (stone) blade is attached to one side and a blunt *boya* edge to the other with *balga* (*Xanthorrhoea* grass tree) resin.

There are no previous studies describing human and weapon efficiency when striking with a hand-held weapon. Here, we focus on two iconic and widespread Aboriginal Australian weapons, one from each side of the continent: the *kodj* from southwestern Australia and the *leangle* and parrying shield from the southeast (Fig. 1). Our biomechanical evaluation of the *kodj* and *leangle* strikes provides a first understanding of the coordinated movement and energy required to use these weapons effectively. In broader terms, this analysis enables an initial understanding of the physical demands required to master these weapons, while offering a wider perspective on combat techniques and cultural practices related to their use.

## Results

**Fig. 2:**
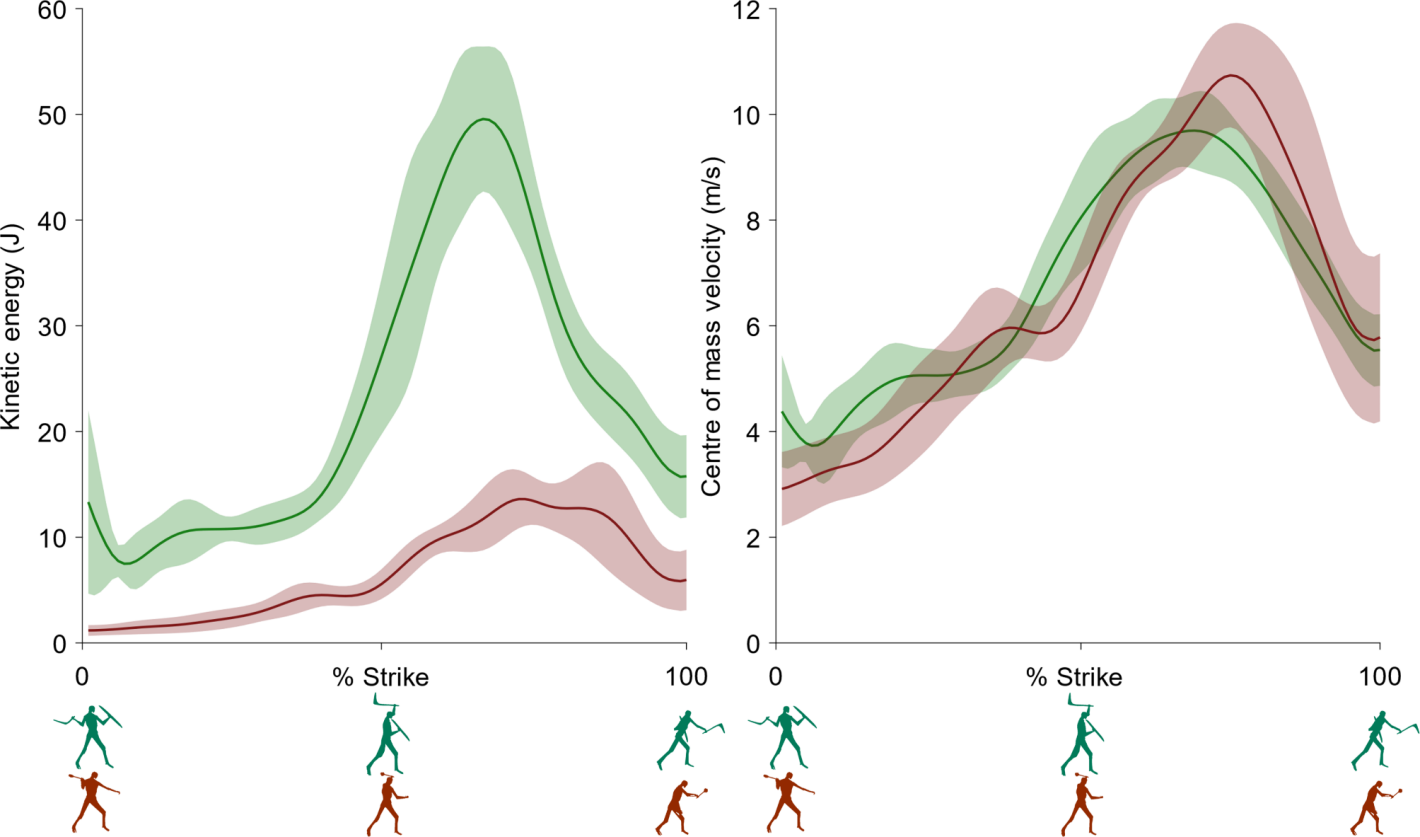
Weapon energy and velocity. Kinetic energy (left) and centre of mass linear velocity (right) of the *leangle* (green) and *kodj* (red) as a percentage of weapon strike movement. Ensemble average (solid line) ±1 standard deviation (shaded area) of 7 *leangle* and 6 *kodj* strikes are shown.

**Fig. 3:**
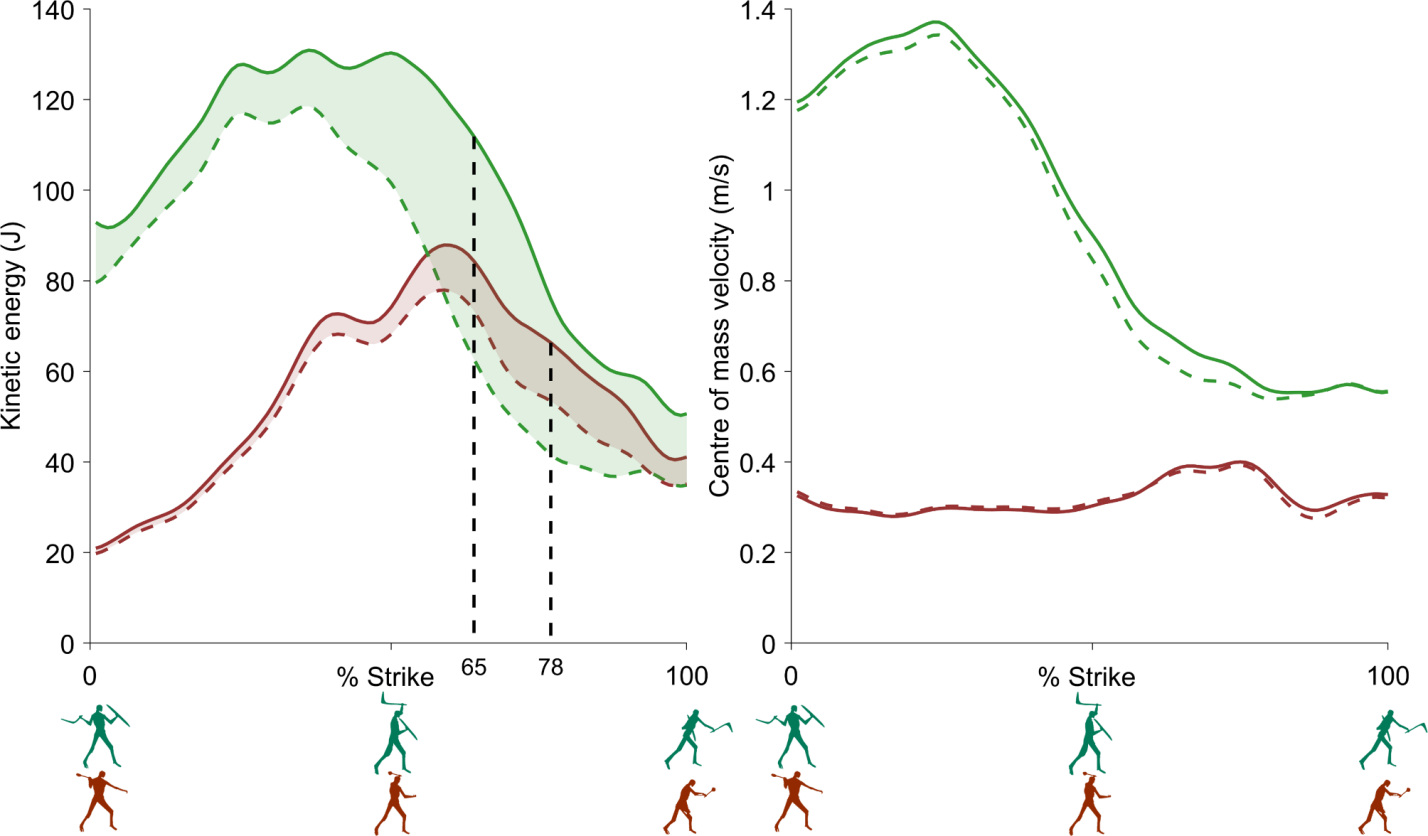
Human energy and velocity. Left: combined body and weapon kinetic energy (solid line), body-only kinetic energy (dashed line), and weapon kinetic energy (shaded area) of the *leangle* (green) and *kodj* (red) as a percentage of weapon strike movement. The vertical dashed lines indicate the time when peak kinetic energy occurred for the *leangle* (65% of strike) and *kodj* (78% of strike). Right: combined body and weapon centre of mass velocity (solid line) and body-only centre of mass velocity (dashed line) of the *leangle* (green) and *kodj* (red) as a percentage of weapon strike. Ensemble average of 7 *leangle* and 6 *kodj* strikes are shown.

**Fig. 4:**
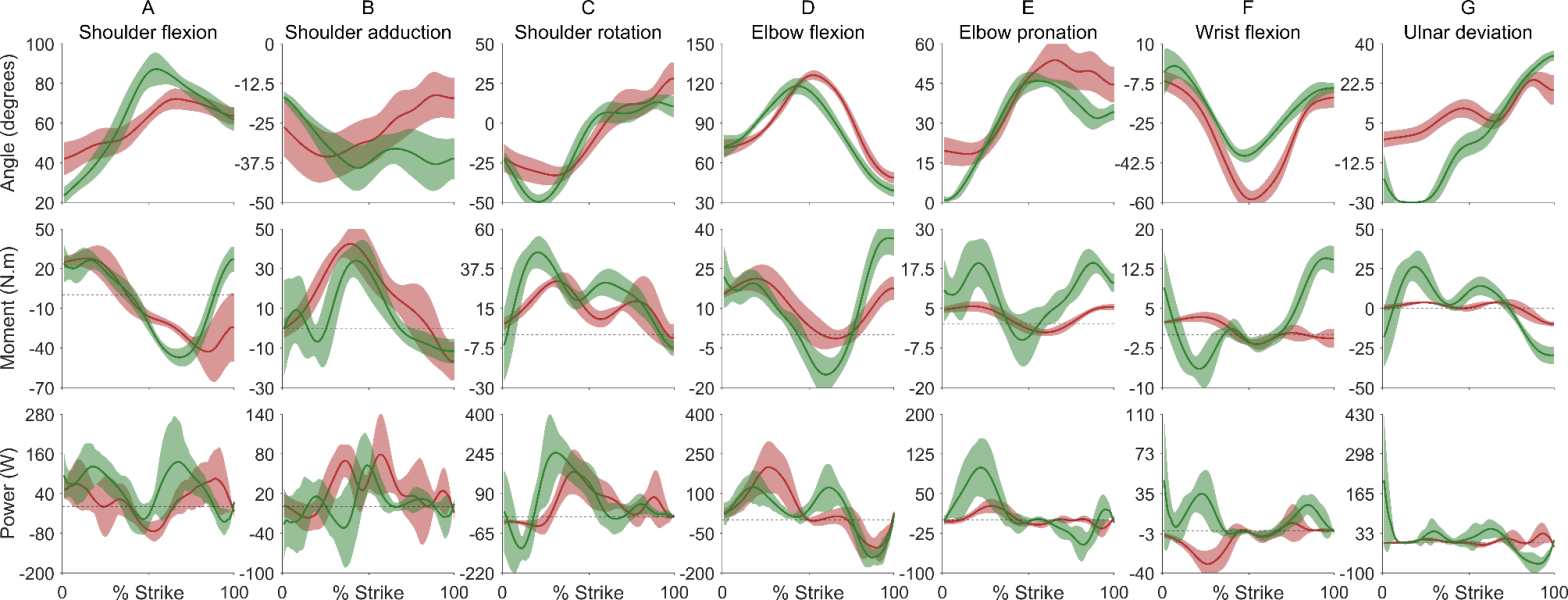
Upper body biomechanics. Angles (top row), moments (middle row), and powers (bottom row) for the shoulder (columns **A**–**C**), elbow (columns **D**, **E**), and wrist (columns **F**, **G**) joints as a percentage of weapon strike movement. Ensemble average (solid line) ±1 standard deviation (shaded area) of 7 *leangle* (green) and 6 *kodj* (red) strikes are shown.

We analysed the mean of seven trials when striking with the *leangle* and the mean of six trials when striking with the *kodj*. One *kodj* trial was omitted from analysis due to missing inertial measurement unit data. The peak kinetic energy of the *leangle* (52.33 ± 7.28 J) was 3.6 times higher than the *kodj* (14.51 ± 3.34 J) (Fig. 2). The peak kinetic energy of the *leangle* occurred within the third quarter of the strike motion (65.43 ± 5.06% strike, Fig. 3) when the weapon reached its maximum velocity (10.04 ± 0.77 m/s, Fig. 2) and had begun its downward trajectory towards the target. The peak kinetic energy of the *kodj* occurred within the final quarter of the strike motion (78.00 ± 6.99% strike, Fig. 3) when the weapon reached is maximum velocity (10.86 ± 0.91 m/s, Fig. 2) and shoulder adduction and elbow pronation were at their near maximums (Fig. 4). The peak velocities of the weapons were comparable (mean difference = 7.6%), but the larger mass of the *leangle* (201% larger than the *kodj*) drove substantial between-weapon differences in peak kinetic energy.

The peak kinetic energy of the body when striking using the *leangle* (145.72 ± 55.40 J) was 1.7 times higher than when striking with the *kodj* (84.33 ± 16.93 J) (Fig. 3). The kinetic energy of the of the total system (person + weapon) during a *leangle* strike (164.25 ± 49.64 J) also far exceeded that of the *kodj* (94.01 ± 17.74 J), but a greater proportion of the *leangle’s* total system kinetic energy (43.44 ± 7.80% vs. 22.85 ± 4.56%) was attributable to the weapon (Fig. 3—shaded region at vertical dotted line corresponding to peak weapon kinetic energy). In contrast to the peak velocities of the weapons which were comparable in magnitude and occurred during the downward trajectory towards the target, the body’s peak velocity when striking with the *leangle* (1.54 ± 0.40 m/s) occurred during the weapon’s initial upward trajectory and was 3.4 times faster than body’s peak velocity when striking with the *kodj* (0.46 ± 0.12 m/s) (Fig. 3).

During the second half of the *leangle* strike (50–100%), the shoulder was moving into more flexion and abduction and the wrist into more ulnar deviation to increase the weapon’s velocity (Fig. 4, row 1-a, b, g). In contrast, the shoulder was moving towards adduction and the elbow into more pronation to increase the *kodj’s* velocity (Fig. 4, row 1-b, e). The wrist flexion and radial deviation moments (Fig. 4, row 2-f, g) and powers (Fig. 4, row 3-f, g) were 3–10 times higher during the final 20% of the *leangle* strike compared to the *kodj* strike.

## Discussion

Clearly, the item made specifically for being an interpersonal weapon (the *leangle*) is far more effective at delivering devastating blows to the human body. In contrast, the *kodj*, being a multi-functional tool primarily used in everyday subsistence, is effective for delivering severe blows that can cause death but is less effective in this function. It is perhaps no surprise the *leangle* possesses a much higher kinetic energy when striking compared to the *kodj*, given its unique shape and larger mass. However, the human kinetic energy requirement during a *leangle* strike is also higher than the *kodj*, but to a much lesser degree. The *leangle* requires its user to generate 75% more energy, but in turn, carries 260% more energy than the *kodj*—with potentially deadly consequences. Given the *kodj* is a multi-functioning tool, it is seemingly appropriate that it is more efficient for a human to manoeuvre (i.e., lower kinetic energy requirement).

Despite its large mass, the *leangle* can reach peak velocities during its downward trajectory comparable to metal-bladed hand-held weapons, like the Chinese kitchen knife during a slashing motion^19^. The power generated in flexion, first by the shoulder and elbow, then by the wrist, propel the *leangle* from an overhead position down to the target. A *kodj* strike instead relies primarily on power generated by the shoulder, not only in flexion but also in rotation. The whole body simultaneously moves from an open to a closed stance as the elbow pronates to lower the *kodj* to the target. This coordinated human movement makes the multi-functional *kodj* effective for striking targets like tree trunks or animals close to the ground.

Identification of the oldest striking weapons is hampered by the vagaries of the archaeological record. The oldest near complete weapons are one-piece wooden spears (javelins) dating to about 400,000 years ago from Schöningen, Germany^20^, but unaltered (and therefore ‘archaeologically invisible’) stones and branches are no doubt the first objects to be used to strike another individual. Similarly, the oldest clubs might be unaltered long bones or sections of branches. As such they would be wholly organic, and only survive to be recorded in exceptional and extremely rare circumstances. Furthermore, if modifications are not made to the natural item and damage is not accrued from use, archaeologists would have no basis on which to identify the piece as a club.

As the *leangle* and parrying shield are made completely of hardwood, the likelihood of such weapons being found in archaeological contexts is extremely rare. Further, the rate of plant decomposition on the Australian continent is higher than on countries closer to the poles, due to comparatively higher rainfall and soil moisture content, and consequently, a multitude of destructive fungi and termites^21^. Indeed, less than 100 pieces of wooden technology have been recovered from Australian archaeological contexts, with most having no associated age (SI Table 1). To date, the oldest wooden artefacts discovered on the Australian continent consists of 25 tools including boomerangs, one-piece spears, digging sticks, and pointed stakes preserved in peat at Wyrie Swamp, South Australia. These items were dated to between 12,398 and 11,270 cal. BP and 10,375–9.628 cal. BP^22,23^. A direct date was also obtained on one of the boomerang fragments: 9,430 ± 150 BP^23^.

For the *kodj*, identification of this weapon in archaeological contexts is hampered by several factors. First, the resin was likely recycled (it can be heated over a fire and removed), the wooden handle would quickly decay, and the stone *boya*—once removed from its constituent parts, may be difficult to recognise as coming from a *kodj*, rather than another similar flaked tool. At this time, the oldest recovered fragment of an axe (albeit the ground-edge variety rather than flaked) in the Australian context dates from 44,000 to 49,000 years old and was recovered from the Kimberley site of Carpenter’s Gap 1, Western Australia^24^.

In recent times on the Australian continent, a range of weapons are recorded as having been used in both inter-and intra-tribal conflict, as well as dispute resolution including ‘trial by ordeal’, whereby an accused individual must face a barrage of projectiles (spears, fighting boomerangs, etc.) unarmed and sometimes with a shield. Such trials often resulted in injuries, and only rarely in death^25,26^. Furthermore, there are several accounts of women being struck with tools or weapons in domestic situations, including with the *kodj*^27^.

Archaeological evidence for interpersonal violence (indications found on human skeletal remains) on the Australian continent is rare. Discovery depends on individuals first having been buried as part of mortuary practices and burial was only one way the deceased were laid to rest in Aboriginal Australia^28–30^. Individuals who were buried need then to be located, adequately excavated, and recorded in order for skeletal trauma—if present—and potential cause of death to be determined and reported. Thus far, the region surrounding the Murray River has yielded the richest source of information owing to being densely populated, has sites with decent bone preservation, and has seen the most disturbance by European development which resulted in comparatively more burial sites being recovered compared to other areas in Australia^26^.

Pardoe has provided the most comprehensive overview of trauma attributed to interpersonal violence in the Australian archaeological record^26^. He groups these injuries into cranial depression fractures, parrying fracture, and pathologies directly attributed to traumatic origin. He described parrying fractures as “transverse breaks of the distal ulna, above the wrist, resulting from raising the arm in defence against attack by a club, digging stick, or the like”^26,31^. These injuries were equally prevalent in men and women (3.9% parrying fractures among + 700 men and 5.0% among + 700 women from skeletal remains examined by^31^) and can be interpreted as “the result of face-to-face combat or as a reflex action to ward off an unexpected or sudden blow”. Pardoe suggests these fractures could result from a direct blow to an unprotected arm or by a glancing blow off a shield, and could reflect domestic violence, between camp fights (including trial by ordeal), as well as formal combat^31^.

Cranial depression fractures are defined by Pardoe as “small, circular to oval depressions in the outer table of the vault typically measuring 20 mm by 15 mm and a few millimetres deep”^26^. These depressions are said to be caused by a sharp blow to the head with a club or stick that collapses the outer part of the skull and leaves a dent. Pardoe reports these fractures are a “common feature of Australian skeletal biology”^26^, and women tend to exhibit these injuries more than men (21.6% of 1069 men suffered dents to the head and 36.1% of 777 women suffered these wounds). The cause of these blows is considered “most likely to be from domestic disputes (within or between the sexes), sham fights, formal battle, or trial by ordeal”^26^ as well as possibly *Sorry Business* in which grieving family and friends often cut and beat themselves on the head as part of funerary practices.

Identification of fatal injuries from conflict is restricted to only two archaeological finds in the Australian context. The first being skeletal remains of an adult male (30–40 years at time of death) with trauma to his cranium and vertebrae, killed some 3677 cal. BP on sand dunes in what is now Narrabeen, northern Sydney^32^. The presence of flaked stone artefacts including three fragments embedded within and between bones indicates this individual was victim of a ritual or payback killing by spearing with what is known as a ‘death spear’. Researchers determined that “a minimum of two spears were used, while the impact puncture on the skull suggest a third weapon—either a barbed spear or club. The unhealed cut mark on the top of the skull is consistent with a peri-mortem stone axe wound”^32^. The healed depressed skull fracture on the Narrabeen man suggests this individual was involved in previous (non-fatal) dispute resolution. The second example is of a male named Kaakutja who perished at 25–35 years of age. Found in northwest New South Wales in Kurnu Baakantji country, Kaakutja suffered fatal injuries caused by sharp-force trauma by hardwood implement. Indeed, Kaakutja exhibited multiple traumas to the cranium, mandible, and post cranial skeleton, with researchers arguing that a boomerang is the most likely deadly weapon used against this man some 745 ± 20 years ago. The rationale being the length and shape of the injury being consistent with boomerang morphology^33^. Our own study would suggest this weapon could have instead been a *leangle*. This point highlights the value of functional insights that can be obtained through biomechanical analysis to test interpretations arrived at in conventional bioarchaeological studies which could otherwise be described only as plausible opinions.

By considering recent ethnohistorical data, killings such as those recorded in southeastern Australia (just described above) may have been a consequence of dispute settlement, ritual fighting, or violent group conflict^7^. Pardoe argued previously that levels of violence evident along the Murray River have the appearance of organised warfare, and that boundary maintenance, population pressure, alliance networks, and warfare characterised the prehistoric society arrayed along the resource-rich riverine corridor, possibly from as early as 7000 years ago^34^ (see also^35^, who similarly argues for consistent patterns of skeletal trauma over the last 7000 years and^36^).

## Conclusion

Cultures around the globe have invested significant time and effort into designing deadly hand-held weaponry. Although design is critical for weapon efficiency, it is the human who must deliver the deadly strike. Our biomechanical evaluation of the *kodj* and *leangle* strikes provides the first understanding of the coordinated movement and energy expenditure required to use these weapons effectively. The *leangle* is far more effective at delivering devastating blows to the human body, while the *kodj*—a multi-functional tool—is more efficient for a human to manoeuvre and still capable of delivering severe blows that can cause death. The biomechanical methods applied here could be implemented to test other archaic weapons from other periods and regions, with considerable potential to move such studies forward and to allow cross comparisons between investigations.

## Methods

### Weapons

The *kodj* (*kodja*,* kadju*,* käoit*,* coccio*) and *leangle* (*langeel*, *lionile*,* Darn-de-wan*) with parrying shield (*mulga*,* malka*,* elaman*) (Fig. 1) used in this study were created by master tool-makers Mr. Larry Blight (*kodj*) and Mr. Brendan Kennedy and Mr. Trevor Kirby (*leangle* and parrying shield). Created for a public science series exploring Indigenous Australian weapon innovation (by *Blackfella Films*), these replica weapons are consistent with those used in their respective communities. The *kodj* was 365 mm long, 114 mm wide, and 46 mm thick. The *leangle* was 656 mm long, 335 mm wide, and 30 mm thick. These dimensions are comparable to known ethnographic ranges reported for the *leangle*^27,37^ and *kodj*^38,39^.

The *leangle* and shield are both made one-piece in hardwood, a raw material widely available (numerous species present) and used for creation of diverse material culture on the Australian continent^40^. The *kodj*, on the other hand, is a composite weapon incorporating a stone axe and stone flat hammer hafted onto a wooden handle using resin^39^. The Menang Noongar expert tool-maker of the *kodj* used in this study, Mr. Larry Blight, provides more insight into the components of this particular technology stating the *kodj* is constructed by attaching a sharpened *boya* (stone) blade on one side and a blunt *boya* edge on the other to a *boorn* (handle) with *balga* (*Xanthorrhoea* grass tree) resin. He specifies the resin is a combination of *balga* resin, *kop* (charcoal), and *yonga* (dried kangaroo faeces), which when combined is known as *biriny* and is coated over the *boya* and *boorn* while hot and plastic before drying to a hard binding.

Both weapons were used to strike at an opponent. The *leangle* is used in hand-to-hand combat and designed such “that the warrior may be enabled to strike round the shield, or *elaman*, of his adversary”^41^ with Etheridge^37^ reporting that “when a combatant wishes to strike side-wise and from himself with a back-handed blow, the round, and not the point, of the *Leonile*, is used”. Brough Smyth^42^ describes the *langeel* as “perhaps the most dangerous of all the weapons of [hand-held offensive weapons]…because of the facility with which the point can be suddenly turned at the moment of striking, [and] is [thus] difficult to avoid”. The *mulga* or parrying shield which accompanies the *leangle* was designed to be narrow (maximising the warrior’s visibility of his opponent) and bow out to a thick convex centre to better parry away blows from the opponent. These shields often featured engraved and painted designed reflecting the community which made the weapon.

Less is reported ethnographically regarding the use of the *kodj* in fighting, though, like the *leangle*, it can be “pivoted by a turn of the wrist so that the blade can cut in any direction”^38^. Breton^41^ describes this tool as one used to cut notches in tree trunks so that hunters could ascend into the high branches and adds that he has “no doubt [they] use them in their wars as well”. Similar early descriptions of its use in hunting are found in King’s observations of the people of King George III Sound, where he describes the *käoit* as being used in “killing seals and other animals by striking them on the head”^43^, while Salvado (quoted in^39^) stated that the *Kodj* was used “to secure game from dead trees, cut footholds on trunks of trees to enable them to climb, fashion their weapons, break the bones of the kangaroo, and other animals in order to extract the marrow for food or to anoint themselves, and a thousand other uses”.

A multi-view three-dimensional digital reconstruction of each weapon was created from a high definition cyberscan (Myriad Studios; myriadstudios.com.au) in Fusion 360 software (Autodesk, CA, USA). The *leangle* was carved from solid hardwood and assumed to have a constant density (1.130 g.cm^−3^), computed from measured mass (1.055 kg) and mesh volume (933.716 cm^3^). The *kodj* density (1.148 g.cm^−3^) was calculated from total measured mass (0.350 kg) and mesh volume (304.949 cm^3^). To better approximate centre of mass and moments of inertia, the *kodj* handle and head densities were calculated independently. Handle mass was calculated using the mesh volume of the extruded handle and the density of dried acacia/wattle (570 g.cm^−3^)^44^. The density of the *kodj* head was assumed constant and calculated from the remaining mass (measured mass − handle mass) and the mesh volume of the head. Centre of mass and moments of inertia (xy, xz, yz) of each weapon were subsequently computed using Fusion 360 proprietary tools.

### Biomechanics

Three-dimensional motion of the upper body joints during a strike, the corresponding moments of force acting about these joints, and subsequent powers produced by these joints can be quantified using data obtained from inertial measurement units (IMU). Integrating these data into a neuromusculoskeletal model of the human body^45^, with consideration of the physical properties of the weapon (e.g., centre of mass, inertia), enables a comprehensive analysis of the human biomechanics involved in striking using the *kodj* and *leangle*.

Biomechanical data were collected from one adult male Aboriginal Australian who used both weapons. The participant had prior experience using the weapons but did not consider himself an ‘expert’. Ethical approval was obtained from the Griffith University Human Research Ethics Committee (GU#2022/665). The participant was informed of the procedures and provided their written informed consent, consistent with the Declaration of Helsinki, prior to participation.

**Fig. 5:**
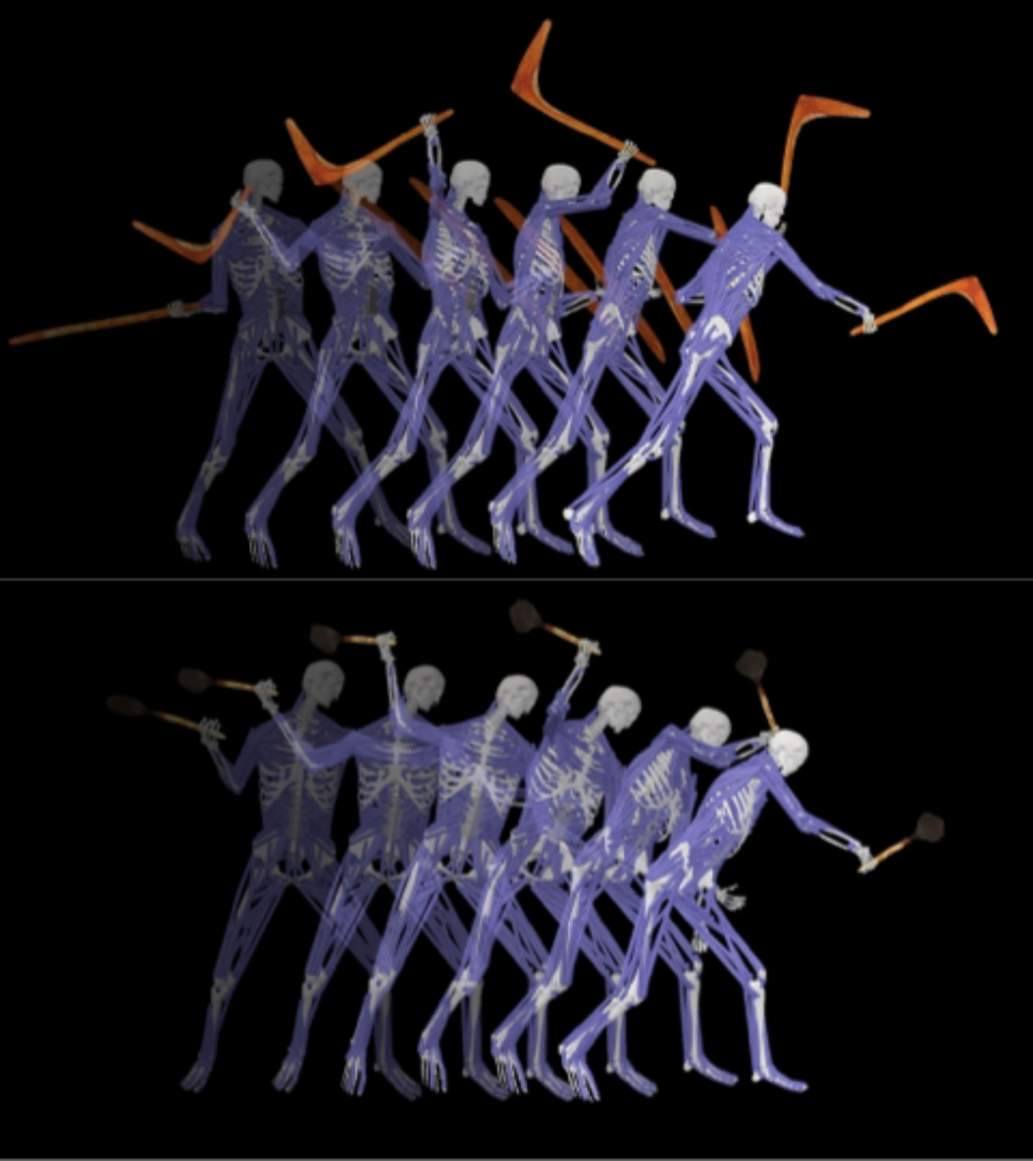
Human biomechanics during *kodj* and *leangle* strikes. Time-lapse depiction of the musculoskeletal system during *leangle* with parrying shield (top) and *kodj* (bottom) strikes.

Seventeen IMU (Xsens/Movella, NV, USA) were attached to the participant’s body. The IMU were placed according to manufacturer guidelines on the head, shoulders, upper arms, forearms, hands, torso, pelvis, thighs, shanks, and feet. Twenty-eight retro-reflective markers (SI Fig. 1) were placed atop bony landmarks and a static trial (i.e., quiet upright stance) of three-dimensional marker locations was record at 200 Hz in Nexus 2.12 (Vicon, Oxford, UK). The participant performed static and functional calibrations to initialise the orientation of the IMU. The *leangle* was used with the parrying shield, and the participant performed seven repeated trials of a striking motion using the *leangle* and then the *kodj* (Fig. 5). All IMU data were recorded at 100 Hz using proprietary software (MVN Analyze, Xsens).

To analyse body kinematics and kinetics, a custom model was constructed in OpenSim version 4.4^45^. A bilateral upper extremity model^46^ with glenohumeral, scapulothoracic, and lumbar motions as well as 35 degrees of freedom was used as a base. The lower extremities were added from a previously validated model^47^, as was a three-axis neck joint. The weapons were attached to the hands of the model and aligned using video recorded concurrent and simultaneous to IMU data. The three-dimensional marker locations were used to linearly scale model dimensions and inertial properties in OpenSim.

The orientations of each body segment and the translations of the pelvis from the reconstructed motion in MVN Analyze were exported and read in MATLAB version 2023a (Mathworks, MA, USA). The orientations of each body segment and the translations of the pelvis were applied to the OpenSim model using inverse kinematics tool with equal tracking weights for all segments of the body. Joint moments were computed with inverse dynamics using OpenSim inverse dynamics tool, during which the input coordinates were low pass filtered with a 6 Hz zero-lag dual-pass 3rd order Butterworth filter. Joint powers were calculated as the product of joint moments and joint angular velocity.

For each trial, the centre of mass velocity was calculated for each segment of the body (e.g., thigh, shank, forearm, head) and weapon in OpenSim. Whole body centre of mass velocity was calculated using a weighted sum, according to:

$${vCOM}_{T}=\frac{\sum_{i=1}^{n}{m}_{i}{v}_{i}}{{m}_{T}}$$

where *m*_*i*_ is the mass of a segment of the body, *v*_*i*_ is the velocity of a segment of the body, *n* is the number of body segments, and *m*_*T*_ is the total mass of all body segments and weapon(s). Kinetic energy was calculated for each weapon and segment of the body according to:

$$KE=0.5*(m{v}^{2}+I{\omega}^{2})$$

where *KE* is kinetic energy, *m* is the mass, *v* is the velocity, *I* is the moment of inertia, and *ω* is the angular velocity. Combined person and weapon (total) kinetic energy was calculated as the sum of all segments of the body and weapon(s). The proportion of weapon kinetic energy with respect to total kinetic energy was also calculated at the timepoint when peak weapon kinetic energy occurred. After calculating joint angles and moments, centre of mass velocity, and kinetic energy, each strike was time-normalised to 101 points to allow for ensemble averaging and between-weapon comparisons. The start of the strike was identified as the timepoint during which the weapon centre of mass was most posterior to the participant’s centre of mass during the backswing. The end of the strike was identified as the first timepoint in which the weapon centre of mass was lower than the elbow.

## Electronic supplementary material

Below is the link to the electronic supplementary material.

Supplementary Material 1

## Data Availability

The data that support the findings of this study are not openly available due to reasons of sensitivity and are available from the corresponding author upon reasonable request. Data are located in controlled access data storage at Griffith University.
