# Supplementary material for: Aboriginal Australian weapons and human efficiency
Source: Sci Rep. 2024 Oct 26;14:25497. doi: 10.1038/s41598-024-76317-w (PMC11513080; doi:10.1038/s41598-024-76317-w)
Supplement: Supplementary file 1 — Supplementary Material 1 [file 41598_2024_76317_MOESM1_ESM.pdf]

Supplementary Information

for

Aboriginal Australian technologies and human efficiency

Laura E. Diamond, Michelle C. Langley, Bradley Cornish, Claudio Pizzolato, David J. Saxby

Including

Figure S1: Position of retro-reflective markers and inertial measurement units on human body for biomechanical assessment.

Table S1: Wooden technology recovered from Australian contexts to date.

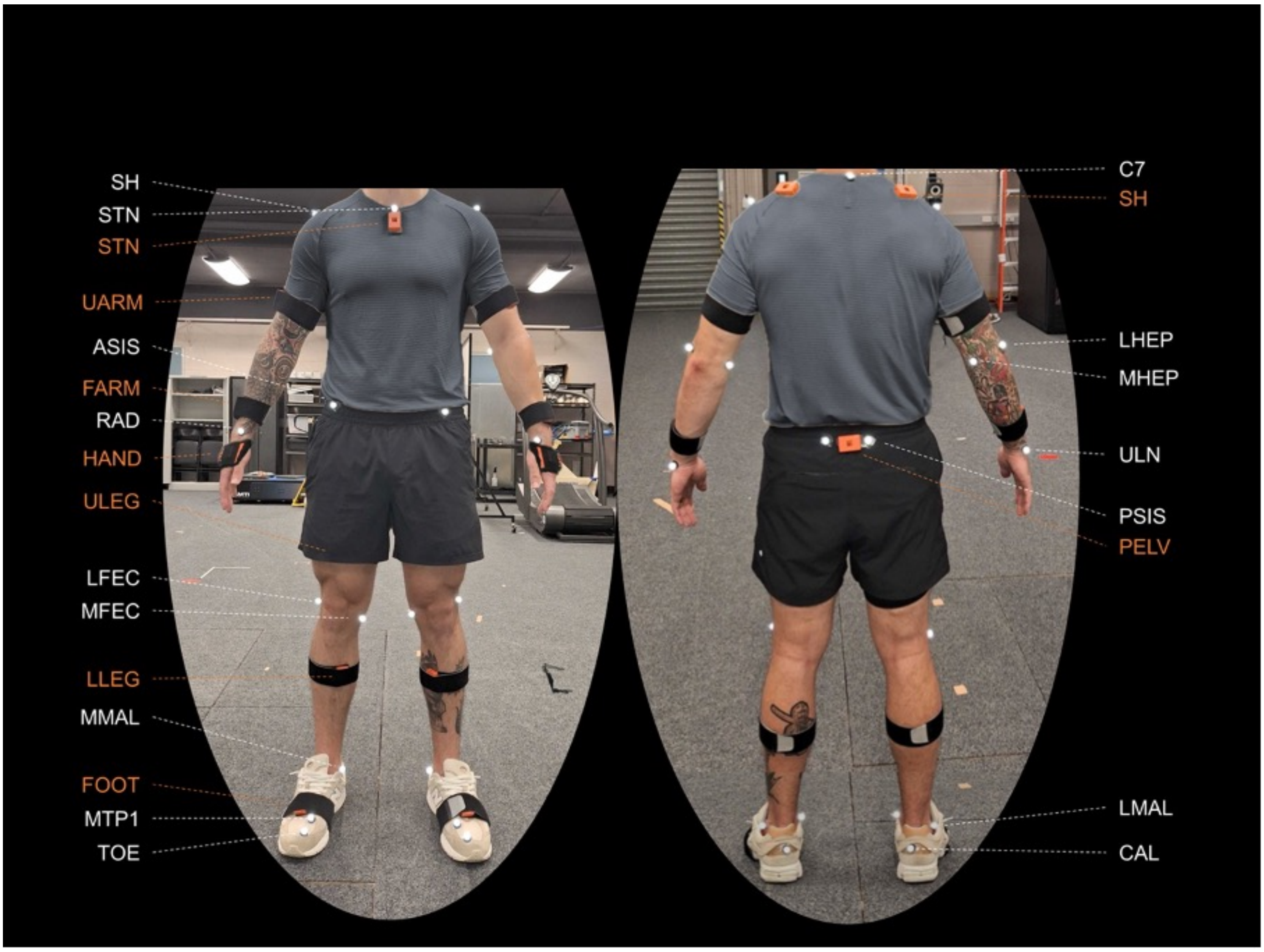

Figure S1. Position of retro-reflective markers (white text) and inertial measurement units (orange text) on human body for biomechanical assessment. **Left:** Anterior view. **Right:** Posterior view. Markers were placed on the sternum (STN), spinous process of the 7th cervical vertebra (C7), and the left and right acromion (SH), anterior superior iliac spine (ASIS), posterior superior iliac spine (PSIS), lateral humeral epicondyle (LHEP), medial humeral epicondyle (MHEP), styloid process of the radius (RAD), styloid process of the ulnar (ULN), lateral femoral epicondyle (LFEC), medial femoral epicondyle (MFEC), medial malleoli (MMAL), lateral malleoli (LMAL), distal phalanx of first toe (TOE), 1st metatarsal phalangeal joint (MTP1), and calcaneus (CAL). Inertial measurement units were placed on the head (not shown), sternum (STN), pelvis (PELV), and left and right shoulder (SH), upper arm (UARM), forearm (FARM), hand, thigh (ULEG), shank (LLEG), and foot. Inertial measurement units were placed on the head (not shown), sternum (STN), pelvis (PELV), and left and right shoulder (SH), upper arm (UARM), forearm (FARM), hand (HAND), thigh (ULEG), shank (LLEG), and foot (FOOT).

Table S1. Wooden technology recovered from Australian contexts to date.

AGE	ARTEFACT/S	SITE	REFERENCE
No date available / Surface Find	Digging stick	Diamantina National Park, QLD	Nugent 2015
No date available / Surface Find	Numerous boomerangs, a throwing stick, hafted adze, a club, a digging stick, a tjurunga.	Mulligan River, QLD	Kelly 1968
No date available / Surface Find	Hafted adze	Wollemi National Park, Blue Mountains, NSW	Kelleher 2009
No date available / Surface Find	Firestick	Wollemi National Park, Blue Mountains, NSW	Nugent 2015
No date available / Surface Find	Boomerang and club	Blue Mountains, NSW	Attenbrow 2009
No date / Contact Period	54 wooden artefacts associated with processing skins - bark slabs and wooden pegs	Victoria Rang, Gariwerd, VIC	Gunn 2009
Less than 1,000 years (based on multiple C14 dates through stratigraphy)	>38 wooden artefacts including wood shavings barbed and unbarbed points, fire sticks	Anbangbang I, NT	Allen and Brockwell 2020; Clarke 1985; Jones and Johnson 1985
No date available	Thousands of wood shavings, some 20 broken fragments of wooden technology including spear points, shafts, fire sticks	Djuwarr 1, NT	Allen and Brockwell 2020; Clarke 1989; Jones and Johnson 1985
No date available	Hafted adze	Argaluk Hill, Site 2, NT	Attenbrow 2008; Setzler and McCarthy 1950
No date / Contact Period	11 wooden implements including points, a message stick, a link shaft, and fire sticks, along with numerous wooden shavings	Parbari Midden, Arnhem, NT	Schrire 1982
Direct date: 224±50 BP (NZA 10301)	Digging stick	Namadgi National Park, ACT	Argue 1995; Argue et al. 2001
Direct date: 281-157 cal. BP (140±70 BP; GaK 1299)	Boomerang	Clarence River, NSW	McBryde 1977
Direct date: 480±70 BP	Boomerang and spear point - possibly from a multi- pronged fishing spear	Trial Bay Creek, NSW	McBryde 1977
Direct date: 670±20BP (S-ANU 43337)	Boomerang tip	Riwi, Kimberleys, WA	Langley et al. 2016
Associated date: 940±60 cal. BP	Digging stick	Mordor Cave, Cape York Peninsula, QLD	David 1992
Associated date: 2,151-1,894 cal.BP (Beta 28188)	Bipoint	Nara Inlet 1, Hook Island, QLD	Barker 1989, 1996, 2004

Associated dates: 12,398-11,270 cal. BP (10,200±150 BP; ANU 1292) and 10,375-9.628 cal. BP (8990±120 BP; ANU 1293); Direct date: 9,430±150 BP (ANU 1490)	25 pieces of wooden technology, including boomerangs, one-piece spears, barbed spear, digging sticks, pointed stakes.	Wyrie Swamp, SA	Luebbers 1975, 1978

References

Allen, H. & Brockwell, S. Archaeology of the recent: Wooden artefacts from Anbangbang 1 and Djuwarr 1, western Arnhem Land. *Aust. Archaeol.* **86**, 147-159 (2020).

Argue, D. Discovery of a possible digging stick in the southeast region of Australia. *Aust. Archaeol.* **41**, 38–40 (1995).

Argue, D., Hope, G. & Saunders, P. Digging stick site, Namadgi National Park, ACT. *Aust. Archaeol.* **53**, 41–42 (2001).

Attenbrow, V. in *The Makers and Making of Indigenous Australian Museum Collections* (Eds Peterson, N. & L. Allen, L.) pp. 472–507 (Melbourne University Publishing, 2008).

Attenbrow, V. In *Blue Mountain Dreaming* (Eds Stockton, E. & Merriman, J.) pp. 105–128 (Blue Mountain Education and Research Trust, 2009).

Barker, B. Nara Inlet 1: A Holocene sequence from the Whitsunday Islands, central Queensland coast. *Q. Archaeol. Res.* **6**, 53–76 (1989).

Barker, B. Maritime hunter-gatherers on the tropical coast: A social model for change. *Temps* **6**, 31–43 (1996).

Barker, B. *The Sea People: Late-Holocene Maritime Specialisation in the Whitsunday Islands, Central Queensland, Terra Australis 20* (Dept. of Archaeology & Natural History, Australian National University, 2004).

Clarke, A. in *Archaeological Research in Kakadu National Park* (ed. Jones, R.) pp. 77–96. (Australian National Parks and Wildlife Service, 1985).

Clarke, A. in *Plants in Australian Archaeology* (eds. Beck, W., Clarke, A. & Head, L.) pp. 54–89. (University of Queensland, 1989).

David, B. Recent research in southeast Cape York: Nurrabullgin and Mordor Cave. *Q. Archaeol. Res.* **9**, 50–53 (1992).

Gunn, R. Wooden artefacts from Gariwerd Rockshelters, Western Victoria. *Aust. Archaeol.* **68**, 23–30 (2009).

Jones, R. & Johnson, I. *Archaeological Research in Kakadu National Park* (Ed. Jones, R) pp. 39–76. (Australian National Parks and Wildlife Service, 1985).

Kelleher, M. in *Blue Mountains Dreaming* (Eds Stockton, E. & Merman, J.) pp. 73–102. (Blue Mountain Eduction and Research Trust, 2009).

Kelly, J. D. Hut sites, rock engravings, stone arrangements, and Tjurunga. Mulligan River, Queensland. *Mankind* **6**, 563–566 (1968).

Langley, M. C. et al. A 600-year-old boomerang fragment from Riwi Cave (South Central Kimberley, Western Australia). *Aust. Archaeol.* **82**, 106-122 (2016).

Luebbers, R. A. Ancient boomerangs discovered in South Australia. *Nature* **253**, 39 (1975).

Luebbers, R. A. *Meals and Menus: A Study of Change in Prehistoric Coastal Settlement in South Australia* (Unpublished PhD thesis, Australian National University, Canberra, 1978).

McBryde, I. Some wooden artefacts from the north coast of NSW: New archaeological and ethnographic data. *Rec. Aust. Mus.* **31**, 660–671 (1977).

Nugent, S.J. *Sticks and Stones: A Functional Analysis of Aboriginal Spears from Northern Australia*. (Unpublished PhD thesis, School of Social Science, The University of Queensland, 2015).

Schrire, C. *The Alligator Rivers: Prehistory and Ecology in Western Arnhem Land. Terra Australis 7* (Dept. of Prehistory, Research School of Pacific Studies, Australian National University, 1982).

Setzler, F. M. & McCarthy, F. D. A unique archaeological specimen from Australia. *J. Washin. Acad. Sci.* **40**, 1–5 (1950).
